# Successful Treatment of Bexarotene-Induced Central Hypothyroidism

**DOI:** 10.1210/jcemcr/luae041

**Published:** 2024-04-01

**Authors:** Marissa Tucci, Robert Galagan, Dragana Lovre

**Affiliations:** Department of Endocrinology, Tulane University School of Medicine, New Orleans, LA 70112, USA; Department of Endocrinology, Tulane University School of Medicine, New Orleans, LA 70112, USA; Southeast Louisiana Veterans Health Care System (SLVHCS), New Orleans, LA 70112, USA; Department of Endocrinology, Tulane University School of Medicine, New Orleans, LA 70112, USA; Southeast Louisiana Veterans Health Care System (SLVHCS), New Orleans, LA 70112, USA

**Keywords:** central hypothyroidism, bexarotene, levothyroxine, liothyronine

## Abstract

The synthetic retinoid bexarotene (BXT), used in the treatment of cutaneous T-cell lymphoma (CTCL), has been associated with central hypothyroidism due to suppression of thyrotropin (TSH) secretion and upregulation of peripheral thyroxine (T4) and triiodothyronine (T3) metabolism. We present a case of a 41-year-old man with CTCL who developed central hypothyroidism within 1 month of receiving BXT. He required sequential uptitration of levothyroxine (LT4) over 15 months, and free T4 (FT4) and total T3 levels were normalized by a daily regimen of LT4 600 mcg and liothyronine (LT3) 15 mcg. While almost all patients regain normal hypothalamic-pituitary-thyroid axis function after cessation of BXT, there are limited data regarding LT4 and LT3 dosing required to adequately treat central hypothyroidism in patients on BXT. Our patient required an LT4 dose approximately 2.8 times the calculated weight-based dose and LT3 supplementation, demonstrating a large LT4/LT3 combination dose may be required to compensate for BXT-induced central hypothyroidism.

## Introduction

Drug-induced central hypothyroidism has been associated with many drugs, including the synthetic retinoid bexarotene (BXT), an effective treatment of cutaneous T-cell lymphoma (CTCL) (1). BXT induces central hypothyroidism by 1) inhibition of the gene promoter for the thyrotropin (TSH) β-subunit and 2) induction of enzymes responsible for peripheral metabolism of thyroxine (T4) and triiodothyronine (T3) (2, 3). While nearly all patients regain thyroid axis integrity with cessation of BXT, there is scant information regarding the management of symptomatic hypothyroidism in patients requiring long-term BXT therapy. We present a patient who was successfully treated for resistant central hypothyroidism in the setting of long-term BXT for CTCL.

## Case Presentation

**Figure 1.**
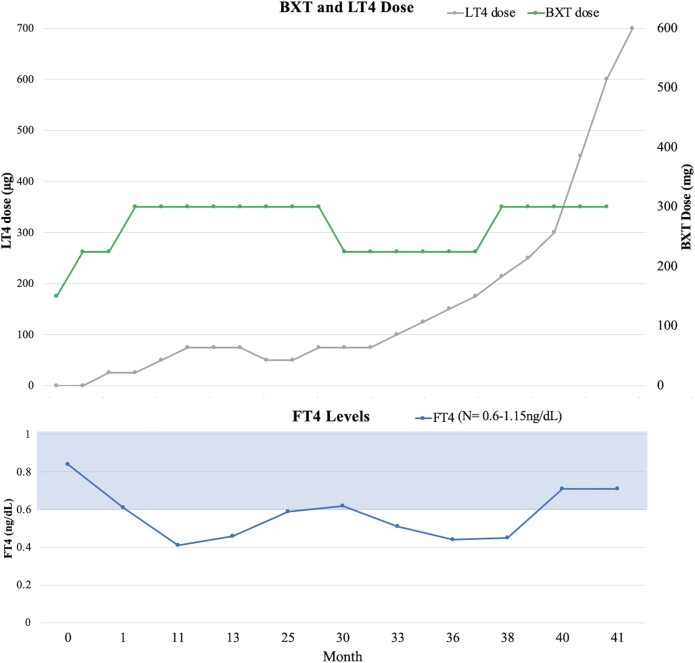
Effect of levothyroxine (LT4) on free thyroxine (FT4) levels in bexarotene (BXT)-induced hypothyroidism.

A 41-year-old man with a medical history of CTCL, obesity (body mass index of 43.65kg/m^2^), hyperlipidemia, and obstructive sleep apnea treated with continuous positive airway pressure presented with worsening fatigue due to treatment-resistant hypothyroidism associated with BXT treatment. Seven months prior to BXT initiation, his thyroid hormone panel was normal: TSH 1.03 mIU/L (1.03 IU/L) (normal 0.3-3.0 μIU/mL [0.3-3.0 IU/L]), free thyroxine (FT4) 0.79 ng/dL (10.17 pmol/L) (normal 0.6-1.15 ng/dL, 7.7-14.8 pmol/L). Two months prior to BXT therapy, his TSH was 0.34 mIU/L (0.34 IU/L) and free T4 (FT4) 0.84 ng/dL (10.82 pmol/L). After 1 month of BXT (225 mg daily), he complained of progressive fatigue and follow-up laboratory values revealed a TSH of 0.17 mIU/L (0.17 IU/L) and FT4 0.61 ng/dL (7.86 pmol/L). After 2 months of BXT (300 mg daily), his FT4 was low at 0.41 ng/dL (5.28 pmol/L) and TSH was low at 0.07 mIU/L (0.07 IU/L), thus levothyroxine (LT4) 25 mcg daily was prescribed. Despite uptitrations to a daily LT4 dose of 125 mcg, he reported persistent fatigue and FT4 and free T3 (FT3) levels remained low at 0.44 ng/dL (5.67 pmol/L) and 1.63 pg/mL (2.52 pmol/L) (normal 2-4.4 pg/mL [3.07-6.14 pmol/L]), respectively (Fig. 1). Endocrinology was consulted for further treatment recommendations.

## Diagnostic Assessment

A review of systems was positive for constipation and difficulty losing weight despite reduced caloric intake and increased physical activity. A review of his medication records was negative for medications known to interfere with LT4 or LT3 absorption. Physical examination revealed normal vital signs, normal thyroid gland by palpation, and a diffuse rash consistent with stage 4 CTCL. Deep tendon reflexes were diminished (1+). A laboratory panel including morning testosterone, luteinizing hormone, prolactin, insulin-like growth factor 1, cortisol, and adrenocorticotropin were within normal limits. Low vitamin B_12_ and 25-hydroxyvitamin D levels were corrected with daily supplements. A celiac panel was negative. A 5-hour LT4 absorption test, administering 1.6 mcg/kg × 7 dose of LT4 (4), resulted in 93% absorption (normal >60%), calculated using the formula: %Absorbed = [[increment total T4 (mcg/dL)×10 (dL/L)]/total administered LT4 (mcg)]] × Vd (L) × 100 (4). A thyroid ultrasound revealed small subcentimeter cysts and nodules, while brain magnetic resonance imaging revealed no hypothalamic or pituitary abnormalities.

Prior to BXT treatment, the patient's lipid measurements were normal, except for decreased high-density lipoprotein of 34 mg/dL (0.88 mmol/L) (normal >40 mg/dL [>1.03 mmol/L]). Three months after BXT initiation, lipid levels increased significantly: Total cholesterol 266 mg/dL (6.89 mmol/L) (normal < 200 mg/dL [<5.17 mmol/L]), triglycerides 296 mg/dL (3.34 mmol/L) (normal <150 mg/dL [<1.69 mmol/L]), and low-density lipoprotein (LDL) 179 mg/dL (4.63 mmol/L) (normal <100 mg/dL [<2.59 mmol/L]). Despite fenofibrate and atorvastatin therapy, elevated cholesterol, triglycerides, and LDL persisted due to his persistent hypothyroidism. Statistical analysis demonstrated an inverse relationship between FT4 and both cholesterol (correlation coefficient −0.62) and triglycerides (correlation coefficient −0.34).

## Treatment

Endocrinology was consulted, and LT4 was rapidly titrated to the patient's weight-based dose of approximately 212 mcg daily. Subsequent laboratory values demonstrated a further decline in FT4 to 0.44 ng/dL (5.67 pmol/L), prompting an increase in LT4 dose to 250 mcg daily. However, FT4 remained low at 0.45 ng/dL (5.80 pmol/L). The patient adhered to taking LT4 on an empty stomach an hour before eating and denied taking vitamins, iron, or other supplements known to interfere with thyroid hormone absorption. To expedite hypothyroidism correction, LT3 was added. The combination of LT4 and LT3 were uptitrated on a bimonthly basis to total daily doses of LT4 600 mcg and LT3 20 mcg when his FT4 finally normalized at 0.71 ng/dL (9.14 pmol/L) and total T3 at 87 ng/dL (133.65 nmol/L) (normal 71-180 ng/dL [0.9 to 2.8 nmol/L]) (see Fig. 1). Lipid levels also improved with total cholesterol at 146 mg/dL (3.7 mmol/L), triglycerides at 195 mg/dL (2.2 mmol/L), high-density lipoprotein at 35 mg/dL (0.91 mmol/L), and LDL at 72 mg/dL (1.86 mmol/L).

## Outcome and Follow-up

On LT4 600 mcg and LT3 20 mcg, both FT4 and total T3 normalized. Subsequently, the LT3 was discontinued and LT4 dose was increased to 700 mcg daily and maintained the FT4 in the normal range 0.71 ng/dL (9.14 pmol/L). On the LT4 dose of 700 mcg daily, the patient reported that he felt well with normal energy.

## Discussion

This case report is unique for several reasons. Our patient required significantly higher LT4 doses to normalize thyroid hormone levels than previously reported in BXT-induced hypothyroidism cases. The effective 700 mcg daily dose of LT4 was approximately 3 times the calculated weight-based dose for thyroid replacement. Prior reports suggested LT4 dosing between 1 and 2 times the expected weight-based dose (125-225 mcg daily) was adequate for patients on BXT 300 to 400 mg daily (3). After excluding malabsorption factors, the main reason for our patient's increased LT4 requirement was BXT's mechanisms of action: 1) inhibition of the gene promoter for the TSH β-subunit, TSH synthesis/secretion, and 2) induction of enzymes responsible for peripheral thyroid hormone metabolism, including type 1 deiodinase activity and hepatic detoxification enzymes (3). Other potential factors contributing less significantly to increased thyroid hormone requirement were metabolic syndrome, obesity (body mass index 43.7kg/m^2^) and type 2 diabetes mellitus.

A study comparing metabolically healthy obese to metabolically unhealthy obese patients demonstrated that both groups had higher FT3 levels and FT3/FT4 ratios (*P* < 0.05), but the metabolically unhealthy obese individuals had significantly lower FT4 (*P* < .05) and higher TSH levels (*P* < .01) (5). Similarly, excess adipose tissues with increased adipokines have been associated with decreased central and peripheral thyroid hormone sensitivity in euthyroid patients (6). Moreover, type 2 diabetes mellitus has been implicated in thyroid hormone resistance (7). Ultimately, these comorbidities likely had a modest effect on the exceedingly high LT4 dose required.

Clinical and biochemical euthyroidism was achieved with LT4/LT3 combination treatment. The role of LT4/LT3 combination treatment of hypothyroidism has been controversial; still, combination LT4 and LT3 replacement therapy may be beneficial in the treatment of resistant and persistently symptomatic hypothyroid patients (8). Our patient's FT3 was low (FT3 1.63 pg/mL, 2.52 pmol/L), which we attribute to the inhibitory effect of BXT on peripheral thyroid hormone metabolism and was corrected with LT3 dosing. Our literature search found only one case of LT4/LT3 combination therapy in the treatment of BXT-induced hypothyroidism and the patient's weight was not reported, therefore we were unable to assess the expected LT4 dose replacement (9). Our patient's response to combination LT4/LT3 therapy highlights the need for more comprehensive research on thyroid hormone replacement in central hypothyroidism.

The patient also experienced elevations in triglycerides and LDL cholesterol resistant to fenofibrate and atorvastatin treatment. Prior to BXT treatment, the patient's total cholesterol, LDL, and triglycerides were normal but quickly increased on BXT, consistent with the medical literature (10). Despite atorvastatin and fenofibrate therapy, his total cholesterol, LDL, and triglyceride levels remained elevated due to his persistent hypothyroidism. The lipid levels improved with normalization of FT4.

Guidelines recommend pretreatment with LT4 in all patients receiving BXT to prevent symptomatic hypothyroidism (11), initiating LT4 25 to 50 mcg daily as soon as BXT is begun with the goal of maintaining FT4 within the upper third of normal range. LT4 and LT3 doses should be adjusted with BXT dosage changes. A retrospective study in Japan (n = 66) demonstrated that prophylactic LT4 treatment was beneficial in preserving euthyroidism in CTCL patients after BXT initiation (12). Monitoring FT4 levels biweekly until within the target range, then monthly, is recommended (2, 13). Patients on pre-BXT LT4 should increase LT4 dosage by 25 to 50 mcg daily and be followed similarly. When BXT is discontinued, LT4 can be stopped concurrently or lowered to pre-BXT dosing. Repeating the thyroid function tests 1 month after BXT discontinuation is reasonable to confirm euthyroidism. Endocrinology consultation should be routine for BXT-induced hypothyroidism.

In summary, we report a case of BXT-induced central hypothyroidism necessitating an LT4 dose approximately 3 times the calculated weight-based dose, along with LT3 supplementation for timely biochemical and clinical euthyroidism. These dosing requirements notably surpass previous reports, illustrating the substantial LT4/LT3 combination dose needed to counter BXT-induced TSH suppression and accelerated peripheral thyroid hormone metabolism.

## Learning Points

- BXT causes central hypothyroidism by suppression of TSH secretion and acceleration of peripheral T4 and T3 metabolism.
- In central hypothyroidism, treatment response is assessed through monitoring FT4 levels rather than TSH, irrespective of the underlying cause.
- BXT also affects other metabolic pathways, such as serum lipids, which may require treatment with lipid-lowering agents.
- Due to the increase in peripheral T4 and T3 metabolism with BXT, prophylactic LT4 treatment should be considered when initiating BXT.
- Compared to the treatment of other causes of hypothyroidism, larger doses LT4 and LT3 should be anticipated for timely correction of BXT-induced central hypothyroidism and more frequent FT4-level monitoring.

## Contributors

All authors made individual contributions to authorship. M.T. constructed the initial manuscript and figure. D.L. and R.G. were involved in the management of the patient and manuscript writing. All authors reviewed and approved the final draft.

## Data Availability

Data sharing is not applicable to this article as no data sets were generated or analyzed during the current study.

## Abbreviations

BXT: bexarotene
CTCL: cutaneous T-cell lymphoma
FT3: triiodothyronine
FT4: free thyroxine
LDL: low-density lipoprotein
LT3: liothyronine
LT4: levothyroxine
T3: triiodothyronine
T4: thyroxine
TSH: thyrotropin
